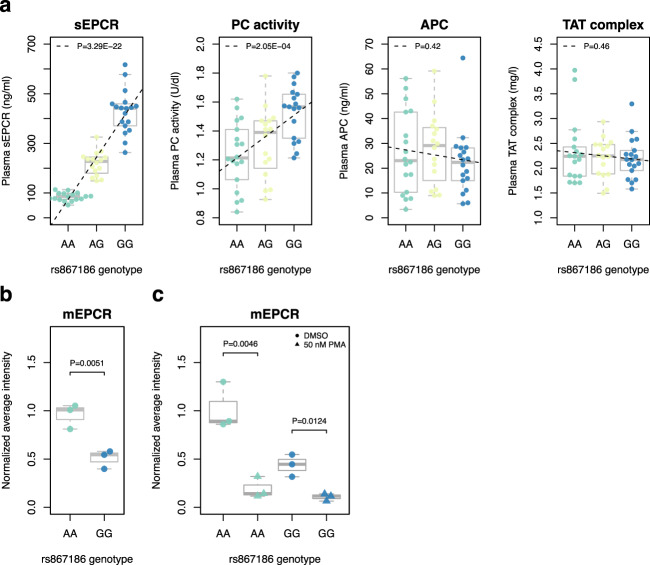# Publisher Correction: Elucidating mechanisms of genetic cross-disease associations at the *PROCR* vascular disease locus

**DOI:** 10.1038/s41467-022-29641-6

**Published:** 2022-04-06

**Authors:** David Stacey, Lingyan Chen, Paulina J. Stanczyk, Joanna M. M. Howson, Amy M. Mason, Stephen Burgess, Stephen MacDonald, Jonathan Langdown, Harriett McKinney, Kate Downes, Neda Farahi, James E. Peters, Saonli Basu, James S. Pankow, Weihong Tang, Nathan Pankratz, Maria Sabater-Lleal, Paul S. de Vries, Nicholas L. Smith, CHARGE Hemostasis Working Group, Abbas Dehghan, Adam S. Heath, Alanna C. Morrison, Alex P. Reiner, Andrew Johnson, Anne Richmond, Annette Peters, Astrid van Hylckama Vlieg, Barbara McKnight, Bruce M. Psaty, Caroline Hayward, Cavin Ward-Caviness, Christopher O’Donnell, Daniel Chasman, David P. Strachan, David A. Tregouet, Dennis Mook-Kanamori, Dipender Gill, Florian Thibord, Folkert W. Asselbergs, Frank W. G. Leebeek, Frits R. Rosendaal, Gail Davies, Georg Homuth, Gerard Temprano, Harry Campbell, Herman A. Taylor, Jan Bressler, Jennifer E. Huffman, Jerome I. Rotter, Jie Yao, James F. Wilson, Joshua C. Bis, Julie M. Hahn, Karl C. Desch, Kerri L. Wiggins, Laura M. Raffield, Lawrence F. Bielak, Lisa R. Yanek, Marcus E. Kleber, Martina Mueller, Maryam Kavousi, Massimo Mangino, Matthew P. Conomos, Melissa Liu, Michael R. Brown, Min-A Jhun, Ming-Huei Chen, Moniek P. M. de Maat, Patricia A. Peyser, Paul Elliot, Peng Wei, Philipp S. Wild, Pierre E. Morange, Pim van der Harst, Qiong Yang, Ngoc-Quynh Le, Riccardo Marioni, Ruifang Li, Scott M. Damrauer, Simon R. Cox, Stella Trompet, Stephan B. Felix, Uwe Völker, Wolfgang Koenig, J. Wouter Jukema, Xiuqing Guo, Amy D. Gelinas, Daniel J. Schneider, Nebojsa Janjic, Nilesh J. Samani, Shu Ye, Charlotte Summers, Edwin R. Chilvers, John Danesh, Dirk S. Paul

**Affiliations:** 1 British Heart Foundation Cardiovascular Epidemiology Unit, Department of Public Health and Primary Care, University of Cambridge, Cambridge, UK; 2 Department of Cardiovascular Sciences, University of Leicester, Leicester, UK; 3 National Institute for Health Research Leicester Biomedical Research Centre, University of Leicester, Leicester, UK; 4 Department of Genetics, Novo Nordisk Research Centre Oxford, Innovation Building, Old Road Campus, Roosevelt Drive, Oxford, UK; 5 Medical Research Council Biostatistics Unit, University of Cambridge, Cambridge, UK; 6 Specialist Haemostasis Unit, Cambridge University Hospitals NHS Foundation Trust, Cambridge, UK; 7 Department of Haematology, University of Cambridge, Cambridge, UK; 8 National Health Service Blood and Transplant, Cambridge, UK; 9 National Institute for Health Research BioResource, University of Cambridge, Cambridge, UK; 10 Department of Medicine, University of Cambridge, Cambridge, UK; 11 Centre for Inflammatory Disease, Department of Immunology and Inflammation, Imperial College London, London, UK; 12 Health Data Research UK London, London, UK; 13 Division of Biostatistics, School of Public Health, University of Minnesota, Minneapolis, MN USA; 14 Division of Epidemiology and Community Health, School of Public Health, University of Minnesota, Minneapolis, MN USA; 15 Department of Laboratory Medicine and Pathology, School of Medicine, University of Minnesota, Minneapolis, MN USA; 16 Genomics of Complex Diseases Group, Sant Pau Biomedical Research Institute, IIB-Sant Pau, Barcelona, Spain; 17 Cardiovascular Medicine Unit, Department of Medicine, Karolinska Institutet, Center for Molecular Medicine, Karolinska University Hospital, Stockholm, Sweden; 18 Human Genetics Center, Department of Epidemiology, Human Genetics, and Environmental Sciences; School of Public Health, The University of Texas Health Science Center at Houston, Houston, TX USA; 19 Department of Epidemiology, School of Public Health, University of Washington, Seattle, WA USA; 20 Seattle Epidemiologic Research and Information Center, Department of Veterans Affairs Office of Research and Development, Seattle, WA USA; 21 Kaiser Permanente Washington Health Research Institute, Seattle, WA USA; 22 SomaLogic Inc, Boulder, CO USA; 23 National Heart and Lung Institute, Imperial College London, London, UK; 24 British Heart Foundation Centre of Research Excellence, University of Cambridge, Cambridge, UK; 25 National Institute for Health Research Blood and Transplant Research Unit in Donor Health and Genomics, University of Cambridge, Cambridge, UK; 26 Health Data Research UK Cambridge, Wellcome Genome Campus and University of Cambridge, Cambridge, UK; 27 Department of Human Genetics, Wellcome Sanger Institute, Hinxton, UK; 28 Department of Epidemiology and Biostatistics, School of Public Health, Imperial College London, London, UK; 29 National Heart Lung and Blood Institute, Division of Intramural Research, Population Sciences Branch, The Framingham Heart Study, Framingham, MA USA; 30 Medical Research Council Human Genetics Unit, Institute of Genetics and Molecular Medicine, University of Edinburgh, Western General Hospital, Edinburgh, UK; 31 Research Unit Molecular Epidemiology, Helmholtz Zentrum München, München, Germany; 32 Department of Clinical Epidemiology, Leiden University Medical Center, Leiden, The Netherlands; 33 Department of Biostatistics, University of Washington, Seattle, WA USA; 34 Cardiovascular Health Research Unit, Department of Medicine, University of Washington, Seattle, WA USA; 35 Office of Research and Development, US Environmental Protection Agency, Chapel Hill, NC USA; 36 Cardiology, VA Boston Healthcare System, Boston, MA USA; 37 Division of Preventive Medicine, Brigham and Women’s Hospital, Boston, MA USA; 38 Population Health Research Institute, St George’s University of London, London, UK; 39 Bordeaux Population Health Research Center, University of Bordeaux, Bordeaux, France; 40 Department of Cardiology, Division of Heart and Lungs, University Medical Center Utrecht, Utrecht University, Utrecht, The Netherlands; 41 Department of Hematology, Erasmus MC University Medical Center, Rotterdam, The Netherlands; 42 Lothian Birth Cohorts, Department of Psychology, University of Edinburgh, Edinburgh, UK; 43 Department of Functional Genomics, University Medicine Greifswald, Greifswald, Germany; 44 Global Health Research, Usher Institute for Population Health Sciences and Informatics, University of Edinburgh, Edinburgh, UK; 45 The Institute for Translational Genomics and Population Sciences, Department of Pediatrics, The Lundquist Institute for Biomedical Innovation at Harbor-UCLA Medical Center, Torrance, CA USA; 46 Massachusetts Veterans Epidemiology Research and Information Center (MAVERIC), VA Boston Healthcare System, Boston, MA USA; 47 Medical Research Council Human Genetics Unit, Institute of Genetics and Cancer, University of Edinburgh, Western General Hospital, Edinburgh, UK; 48 Department of Pediatrics, University of Michigan, CS Mott Children’s Hospital, Ann Arbor, MI USA; 49 Department of Genetics, University of North Carolina at Chapel Hill, Chapel Hill, NC USA; 50 Department of Epidemiology, School of Public Health, University of Michigan, Ann Arbor, MI USA; 51 Department of Medicine, Johns Hopkins University School of Medicine, Baltimore, MD USA; 52 SYNLAB MVZ für Humangenetik Mannheim, Mannheim, Germany; 53 Department of Epidemiology, Erasmus Medical Center, University Medical Center Rotterdam, Rotterdam, The Netherlands; 54 Department of Twin Research and Genetic Epidemiology, Kings College London, London, UK; 55 Population Sciences Branch, National Heart, Lung, and Blood Institute, Framingham, MA USA; 56 Department of Biostatistics, The University of Texas MD Anderson Cancer Center, Houston, TX USA; 57 Department of Cardiology, Cardiology I, University Medical Center, Johannes Gutenberg University Mainz, Mainz, Germany; 58 Hematology Laboratory, La Timone University Hospital of Marseille, Marseille, France; 59 Department of Cardiology, University Medical Center Utrecht, Utrecht, The Netherlands; 60 Department of Biostatistics, Boston University School of Public Health, Boston, MA USA; 61 Centre for Genomic and Experimental Medicine, Institute of Genetics and Molecular Medicine, University of Edinburgh, Edinburgh, UK; 62 Department of Surgery, Perelman School of Medicine, University of Pennsylvania, Philadelphia, PA USA; 63 Department of Psychology, University of Edinburgh, Edinburgh, UK; 64 Section of Gerontology and Geriatrics, Department of Internal Medicine, Leiden University Medical Center, Leiden, The Netherlands; 65 Department of Internal Medicine B, University Medicine Greifswald, Greifswald, Germany; 66 DZHK (German Centre for Cardiovascular Research), Partner Site Munich Heart Alliance, Munich, Germany; 67 Department of Cardiology, Leiden University Medical Center, Leiden, The Netherlands

**Keywords:** Medical genomics, Cardiovascular genetics

Correction to: *Nature Communications* 10.1038/s41467-022-28729-3, published online 09 March 2022.

The original version of this Article contained an error in Fig. 4, in which the label “c” was missing. This has been corrected in both the PDF and HTML versions of the Article.