# Supplementary material for: Elucidating mechanisms of genetic cross-disease associations at the PROCR vascular disease locus
Source: Nat Commun. 2022 Mar 9;13:1222. doi: 10.1038/s41467-022-28729-3 (PMC8907312; doi:10.1038/s41467-022-28729-3)
Supplement: Supplementary file 1 — Supplementary Information [file 41467_2022_28729_MOESM1_ESM.pdf]

# SUPPLEMENTARY INFORMATION

## Supplementary Tables

Table 1. Binding specificity of protein C SOMAmer reagents........................................2
Table 2. Selection of instrumental variables for Mendelian randomization analyses ......3
Table 3. Summary of the results from Mendelian randomization analyses.....................4
Table 4. Characteristics of the participants in the *PROCR*-p.S219G recall-by-genotype study ..........................................................................................5

## Supplementary Figures

Figure 1. Protein C levels and risk of cardiovascular diseases.......................................6
Figure 2. Results from Mendelian randomization analyses .............................................8
Figure 3. Gene expression profiles of *PROCR*, *ITGB2* and *ITGAM* .............................10
Figure 4. EPCR monoclonal antibody testing for flow cytometry ..................................11
Figure 5. Quantification of surface EPCR on HUVECs ................................................11
Figure 6. Quantification of surface EPCR on U937 cells...............................................12
Figure 7. Gating strategy for distinguishing monocytes and neutrophils from FACS-lysed whole blood..................................................................................12
Figure 8. Quantification of surface EPCR on monocytes and neutrophils .....................13
Figure 9. Leukocyte–endothelial cell adhesion in the presence of anti-Mac-1 antibody and sEPCR .........................................................................14
Figure 10. Power calculations for the recall-by-genotype study .....................................15

**Supplementary Table 1. Binding specificity of protein C SOMAmer reagents.** We measured the binding activity ($K_d$ value) of the three pivotal SOMAmer reagents targeting protein C to each of the commercially available proteins, i.e. protein C, APC, sEPCR, thrombin, factor VIIa, protein S, factor V and thrombomodulin. The SOMAmers were '2961-1_2' (annotated as 'Protein C'), '3758-63_3' (annotated as 'APC') and '3758-63_3' (annotated as 'APC'). We confirmed that '2961-1_2' binds both protein C and APC while SOMAmers '3758-63_3' and '3758-68_3' specifically bind APC. None of the three SOMAmers showed binding activity for the other proteins tested.

We then tested whether sEPCR and thrombomodulin interfere with the SOMAmer reagent binding to protein C or whether sEPCR, protein S and factor VIIa interfere with the SOMAmer reagents binding to APC. The results indicate sEPCR and thrombomodulin do not compete with '2961-1_2' for binding to protein C, and sEPCR, protein S and factor VIIa do not compete with '2961-1_2', '3758-63_3' or '3758-68_3' for binding to APC.

<table>
<tr><th></th><th>2961-1_2</th><th>3758-63_3</th><th>3758-68_3</th></tr>
<tr><td>Protein C</td><td>$2.3\times10^{-8}$ M</td><td>> $1.0\times10^{-7}$ M</td><td>> $1.0\times10^{-7}$ M</td></tr>
<tr><td>APC</td><td>$2.1\times10^{-8}$ M</td><td>$1.2\times10^{-8}$ M</td><td>$6.8\times10^{-9}$ M</td></tr>
<tr><td>sEPCR</td><td colspan="3" rowspan="6">no binding at protein concentrations up to $1.0\times10^{-7}$ M</td></tr>
<tr><td>Thrombin</td></tr>
<tr><td>Factor VIIa</td></tr>
<tr><td>Protein S</td></tr>
<tr><td>Factor V</td></tr>
<tr><td>Thrombomodulin</td></tr>
</table>

**Supplementary Table 2. Selection of instrumental variables for Mendelian randomization analyses.** The tables provides further details on the instrumental variables that we selected for the construction of a multi-allelic genetic score at the *PROCR* region to estimate the causal associations between PC/APC and cardiovascular outcomes (i.e. CAD and DVT) (Methods). Association statistics including effect estimates, standard errors and *P*-values were obtained from the published GWAS datasets. Further information on the data sources for these analyses are provided in Supplementary Data 1. Abbreviations: chr, chromosome; SE, standard error; F-stat, F-statistic; EAF, effect allele frequency. [1]Causal direction estimated by MR-Steiger method. [2]Outlier identified by MR-PRESSO. The SNP rs112928119 was identified as an outlier when estimating causal relationship with PE/VTE/DVT.

| SNP | Chr | Position (GRCh37) | Effect allele | Other allele | Exposure | Units | Sample size | EAF | β-value | SE | *P*-value | F-stat | Correct causal direction[1] | Outlier[2] |
|---|---|---|---|---|---|---|---|---|---|---|---|---|---|---|
| rs867186 | 20 | 33764554 | A | G | APC | SD | 3,301 | 0.914 | -0.256 | 0.044 | 8.71E-09 | 33 | TRUE | FALSE |
| rs112928119 | 20 | 33726451 | C | T | PC | SD | 8,911 | 0.773 | 0.135 | 0.010 | 3.93E-40 | 176 | TRUE | TRUE |
| rs145578591 | 20 | 33970133 | G | A | PC | SD | 8,911 | 0.982 | -0.382 | 0.034 | 1.54E-29 | 127 | TRUE | FALSE |
| rs146348076 | 20 | 32526802 | T | A | PC | SD | 8,911 | 0.976 | -0.345 | 0.029 | 8.00E-33 | 142 | TRUE | FALSE |
| rs148316982 | 20 | 35367029 | A | C | PC | SD | 8,911 | 0.978 | -0.209 | 0.032 | 1.05E-10 | 42 | TRUE | FALSE |
| rs17332951 | 20 | 32764183 | T | C | PC | SD | 8,911 | 0.951 | -0.314 | 0.020 | 3.59E-57 | 254 | TRUE | FALSE |
| rs182198088 | 20 | 33628212 | T | C | PC | SD | 8,911 | 0.991 | -0.411 | 0.046 | 6.86E-19 | 79 | TRUE | FALSE |
| rs185595411 | 20 | 34222491 | G | T | PC | SD | 8,911 | 0.986 | -0.428 | 0.038 | 5.99E-29 | 125 | TRUE | FALSE |
| rs190284633 | 20 | 32833621 | G | A | PC | SD | 8,911 | 0.990 | -0.340 | 0.043 | 4.57E-15 | 61 | TRUE | FALSE |
| rs2425068 | 20 | 34214723 | T | C | PC | SD | 8,911 | 0.941 | -0.206 | 0.023 | 2.31E-19 | 81 | TRUE | FALSE |
| rs34140587 | 20 | 32506150 | C | G | PC | SD | 8,911 | 0.392 | 0.059 | 0.009 | 1.60E-11 | 45 | TRUE | FALSE |
| rs4911468 | 20 | 33694254 | A | T | PC | SD | 8,911 | 0.320 | -0.054 | 0.010 | 1.38E-08 | 32 | TRUE | FALSE |
| rs56317999 | 20 | 35386235 | G | A | PC | SD | 8,911 | 0.972 | -0.176 | 0.029 | 1.20E-09 | 37 | TRUE | FALSE |
| rs6088887 | 20 | 34171324 | G | A | PC | SD | 8,911 | 0.807 | 0.071 | 0.011 | 4.45E-11 | 43 | TRUE | FALSE |
| rs6120993 | 20 | 34185161 | G | T | PC | SD | 8,911 | 0.860 | -0.069 | 0.012 | 2.94E-08 | 31 | TRUE | FALSE |
| rs7263119 | 20 | 32254831 | G | T | PC | SD | 8,911 | 0.517 | 0.049 | 0.009 | 1.32E-08 | 32 | TRUE | FALSE |
| rs7271630 | 20 | 32449398 | T | C | PC | SD | 8,911 | 0.840 | -0.107 | 0.012 | 6.89E-20 | 83 | TRUE | FALSE |
| rs73105074 | 20 | 33213192 | A | C | PC | SD | 8,911 | 0.959 | 0.146 | 0.025 | 2.79E-09 | 35 | TRUE | FALSE |
| rs867186 | 20 | 33764554 | A | G | PC | SD | 8,911 | 0.904 | -0.455 | 0.014 | 1.00E-200 | 1,080 | TRUE | FALSE |
| rs906466 | 20 | 35048632 | G | A | PC | SD | 8,911 | 0.807 | -0.073 | 0.011 | 1.52E-11 | 46 | TRUE | FALSE |

**Supplementary Table 3. Summary of the results from Mendelian randomization analyses.** We applied the Wald's ratio method when only 1 instrumental variant was available and the inverse-variance weighted (IVW) method in a multiplicative random-effect meta-analysis framework when >3 instrumental variants were available. We also applied the MR-Egger regression and MR-PRESSO to estimate causal effects. Further, we performed several sensitivity analyses to assess the robustness of our results to potential violations of the MR assumptions (Methods). β-values (causal effect estimates), odds ratios and *P*-values were derived the different MR methods. Q-statistic and respective *P*-values are shown from the Cochran's Q-test for heterogeneity. Egger-intercept and respective *P*-values are provided for horizontal pleiotropy by MR-Egger. Abbreviations: IVW, inverse-variance weighting; SE, standard error; *P*-val, *P*-value; CI, confidence interval; N, number; OR, odds ratio.

***Protein C → Cardiovascular outcomes:***

| Exposure | Outcome | N (SNPs) | MR Wald's Ratio/IVW | | | | MR-Egger | | | | MR-PRESSO | | |
|---|---|---|---|---|---|---|---|---|---|---|---|---|---|
| | | | *β-val (SE)* | *OR [95% CI]* | *P-val* | *Q-stat (P-val)* | *β-val (SE)* | *OR [95% CI]* | *P-val* | *Egger intercept (P-val)* | *β-val (SE)* | *OR [95% CI]* | *P-val* |
| PC | CAD | 19 | -0.13 (0.013) | 0.88 [0.86,0.90] | 4.17E-24 | 17.24 (0.51) | -0.14 (0.018) | 0.87 [0.84,0.90] | 7.42E-14 | 0.002 (0.44) | -0.13 (0.012) | 0.88 [0.86,0.90] | 5.27E-09 |
| PC | Stroke | 18 | -0.11 (0.023) | 0.90 [0.86,0.94] | 2.86E-06 | 13.79 (0.68) | -0.12 (0.034) | 0.89 [0.83,0.95] | 3.94E-04 | 0.003 (0.59) | -0.11 (0.020) | 0.90 [0.86,0.94] | 7.26E-05 |
| PC | Ischemic stroke | 18 | -0.10 (0.025) | 0.90 [0.86,0.95] | 3.77E-05 | 12.48 (0.77) | -0.10 (0.037) | 0.90 [0.84,0.97] | 4.70E-03 | 0.0003 (0.96) | -0.10 (0.021) | 0.90 [0.87,0.94] | 1.64E-04 |
| PC | Large-artery stroke | 18 | -0.06 (0.064) | 0.94 [0.83,1.07] | 3.76E-01 | 15.86 (0.53) | -0.05 (0.096) | 0.95 [0.79,1.15] | 5.98E-01 | -0.001 (0.93) | -0.06 (0.062) | 0.94 [0.84,1.07] | 3.72E-01 |
| PC | Cardioembolic stroke | 18 | -0.16 (0.051) | 0.85 [0.77,0.94] | 2.10E-03 | 17.83 (0.40) | -0.25 (0.075) | 0.78 [0.67,0.90] | 7.32E-04 | 0.019 (0.09) | -0.16 (0.051) | 0.85 [0.77,0.94] | 6.85E-03 |
| PC | Small-vessel stroke | 18 | -0.06 (0.066) | 0.94 [0.82,1.07] | 3.32E-01 | 21.24 (0.22) | 0.05 (0.094) | 1.05 [0.88,1.27] | 5.84E-01 | -0.023 (0.10) | -0.06 (0.066) | 0.94 [0.82,1.07] | 3.46E-01 |
| PC | DVT | 18 | 0.30 (0.037) | 1.34 [1.25,1.44] | 8.70E-16 | 14.21 (0.65) | 0.36 (0.053) | 1.44 [1.30,1.59] | 4.53E-12 | -0.016 (0.07) | 0.30 (0.034) | 1.34 [1.26,1.43] | 9.76E-08 |
| PC | PE | 18 | 0.16 (0.052) | 1.17 [1.06,1.29] | 2.65E-03 | 19.01 (0.33) | 0.24 (0.071) | 1.27 [1.11,1.46] | 6.91E-04 | -0.019 (0.10) | 0.16 (0.052) | 1.17 [1.06,1.29] | 7.97E-03 |
| PC | VTE | 18 | 0.22 (0.032) | 1.24 [1.17,1.32] | 1.05E-11 | 17.80 (0.40) | 0.28 (0.045) | 1.33 [1.22,1.45] | 2.10E-10 | -0.015 (0.04) | 0.22 (0.032) | 1.24 [1.17,1.32] | 3.10E-06 |
| APC | CAD | 1 | -0.22 (0.033) | 0.80 [0.75,0.85] | 9.01E-12 | NA | NA | NA | NA | NA | NA | NA | NA |
| APC | Stroke | 1 | -0.19 (0.062) | 0.83 [0.73,0.94] | 2.76E-03 | NA | NA | NA | NA | NA | NA | NA | NA |
| APC | Ischemic stroke | 1 | -0.19 (0.068) | 0.83 [0.72,0.95] | 5.60E-03 | NA | NA | NA | NA | NA | NA | NA | NA |
| APC | Large-artery stroke | 1 | -0.11 (0.180) | 0.90 [0.63,1.28] | 5.54E-01 | NA | NA | NA | NA | NA | NA | NA | NA |
| APC | Cardioembolic stroke | 1 | -0.38 (0.138) | 0.68 [0.52,0.89] | 5.42E-03 | NA | NA | NA | NA | NA | NA | NA | NA |
| APC | Small-vessel stroke | 1 | -0.10 (0.163) | 0.90 [0.65,1.24] | 5.28E-01 | NA | NA | NA | NA | NA | NA | NA | NA |
| APC | DVT | 1 | 0.62 (0.099) | 1.87 [1.54,2.26] | 2.74E-10 | NA | NA | NA | NA | NA | NA | NA | NA |
| APC | PE | 1 | 0.45 (0.132) | 1.58 [1.22,2.04] | 5.56E-04 | NA | NA | NA | NA | NA | NA | NA | NA |
| APC | VTE | 1 | 0.50 (0.084) | 1.66 [1.40,1.95] | 1.92E-09 | NA | NA | NA | NA | NA | NA | NA | NA |

***Cardiovascular outcomes → protein C:***

| Exposure | Outcome | N (SNPs) | MR IVW | | | | MR-PRESSO | | |
|---|---|---|---|---|---|---|---|---|---|
| | | | *β-val (SE)* | *OR [95% CI]* | *P-val* | *Q-stat (P-val)* | *β-val (SE)* | *OR [95% CI]* | *P-val* |
| CAD | PC | 157 | -0.01 (0.017) | 0.99 [0.95, 1.02] | 0.410 | 168.93 (0.23) | -0.01 (0.017) | 0.99 [0.95, 1.02] | 0.410 |
| VTE | PC | 20 | 0.05 (0.073) | 1.05 [0.91, 1.21] | 0.497 | 19.78 (0.41) | 0.05 (0.071) | 1.05 [0.92, 1.21] | 0.476 |
| DVT | PC | 21 | 0.15 (0.075) | 1.16 [1.00, 1.34] | 0.050 | 15.04 (0.77) | 0.15 (0.064) | 1.16 [1.02, 1.32] | 0.030 |
| CAD | APC | 138 | -0.01 (0.049) | 0.99 [0.90,1.09] | 0.837 | 144.15 (0.32) | -0.01 (0.049) | 0.99 [0.90,1.09] | 0.837 |
| VTE | APC | 19 | -0.06 (0.045) | 0.94 [0.86,1.02] | 0.157 | 22.71 (0.20) | -0.06 (0.045) | 0.94 [0.86,1.02] | 0.174 |
| DVT | APC | 17 | -0.06 (0.037) | 0.94 [0.87,1.01] | 0.101 | 18.23 (0.31) | -0.06 (0.037) | 0.94 [0.87,1.01] | 0.120 |

**Supplementary Table 4. Characteristics of the participants in the *PROCR*-p.S219G recall-by-genotype study.** Characteristics of healthy volunteers are presented as mean and standard deviation (in brackets) or percentage, as appropriate. Continuous and categorical variables between homozygous groups were compared using the two-sample t-test and chi-square test, respectively. Abbreviations: DSP, diastolic blood pressure; HR, heart rate; SBP, systolic blood pressure.

| | **rs867186-AA** | **rs867186-AG** | **rs867186-GG** | ***P*-value** |
|---|---|---|---|---|
| Number of participants | 18 | 16 | 18 | – |
| Female (%) | 33.3 | 31.2 | 33.3 | 0.60 |
| Current smokers (%) | 0 | 0 | 0 | – |
| Family history (%) | 66.7 | 81.2 | 77.8 | – |
| Age (year) | 49.6 (11.4) | 49.6 (8.0) | 49.9 (10.6) | 0.93 |
| Height (cm) | 173.9 (9.2) | 173.0 (8.5) | 175.3 (8.4) | 0.64 |
| Weight (kg) | 76.1 (12.5) | 76.6 (11.2) | 78.1 (11.4) | 0.57 |
| BMI (kg/m$^2$) | 25.1 (3.4) | 25.6 (3.1) | 25.4 (2.6) | 0.78 |
| Body fat (%) | 24.6 (8.8) | 23.6 (9.5) | 24.2 (8.5) | 0.89 |
| SBP, seated (mmHg) | 125.6 (14.7) | 127.6 (15.4) | 126.5 (17.8) | 0.85 |
| DBP, seated (mmHg) | 75.0 (11.5) | 80.1 (9.4) | 78.0 (12.8) | 0.38 |
| HR, seated (beats/min) | 69.6 (20.6) | 64.9 (9.6) | 65.4 (11.8) | 0.44 |
| SBP, standing (mmHg) | 123.6 (12.5) | 130.7 (17.9) | 125.6 (16.5) | 0.65 |
| DBP, standing (mmHg) | 78.0 (12.0) | 82.6 (9.9) | 81.5 (10.7) | 0.29 |
| HR, standing (beats/min) | 74.3 (13.1) | 72.8 (11.6) | 72.8 (14.3) | 0.75 |

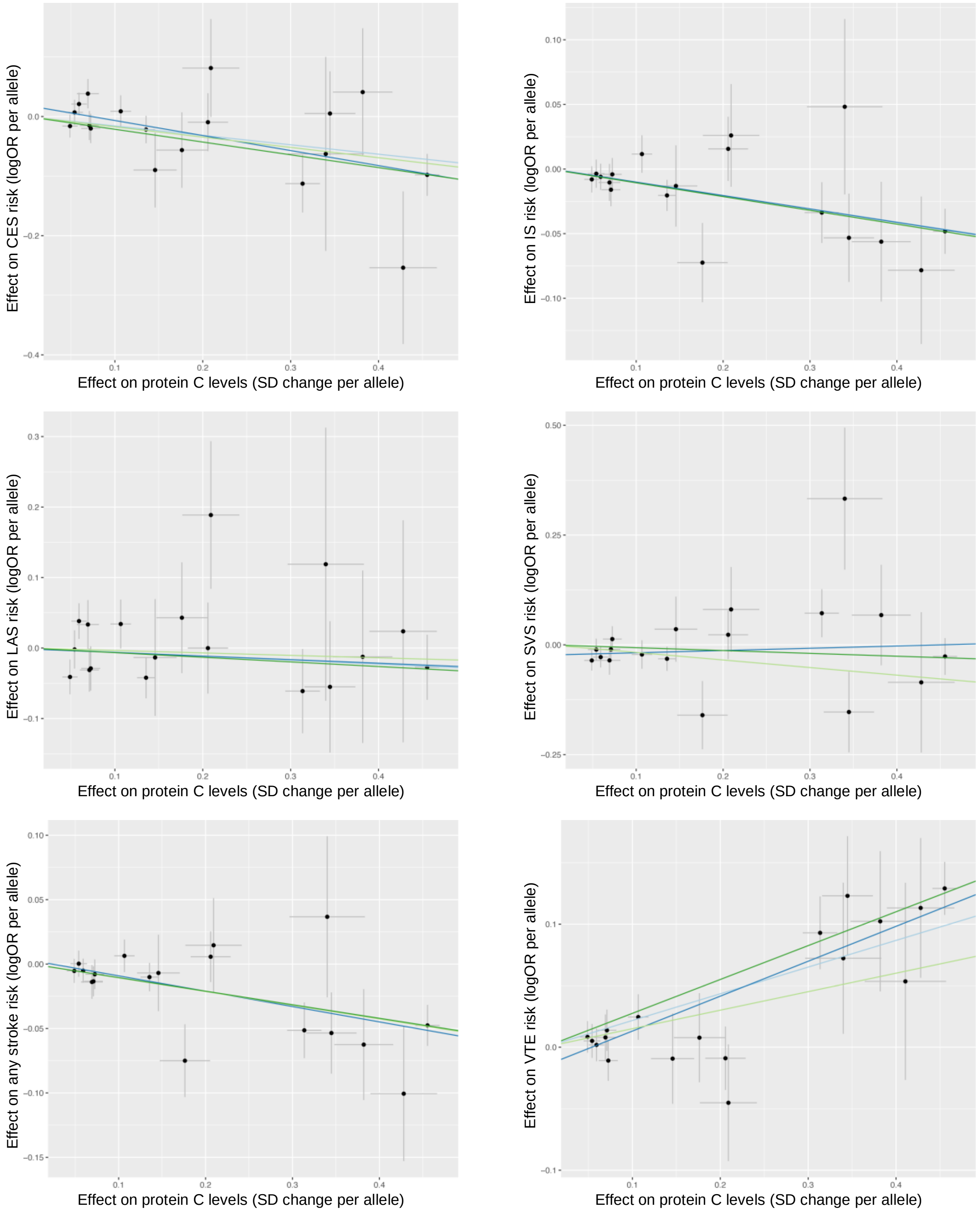
Effect on CES risk (logOR per allele)
Effect on protein C levels (SD change per allele)
Effect on IS risk (logOR per allele)
Effect on protein C levels (SD change per allele)
Effect on LAS risk (logOR per allele)
Effect on protein C levels (SD change per allele)
Effect on SVS risk (logOR per allele)
Effect on protein C levels (SD change per allele)
Effect on any stroke risk (logOR per allele)
Effect on protein C levels (SD change per allele)
Effect on VTE risk (logOR per allele)
Effect on protein C levels (SD change per allele)

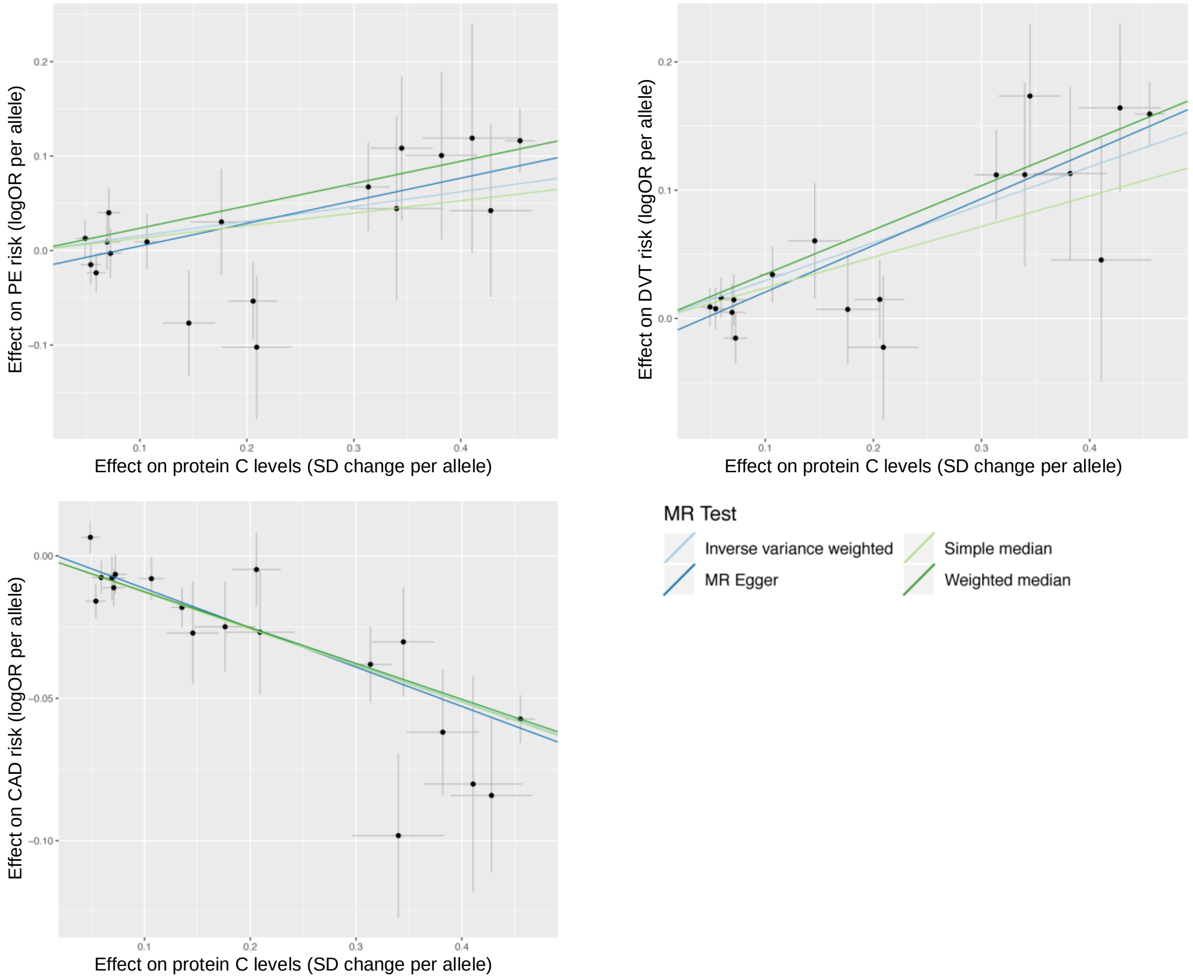


**Supplementary Figure 1. Protein C levels and risk of cardiovascular diseases.** Scatter plot showing the estimated effect sizes and 95% confidence intervals on plasma protein C levels (from linear regression; horizontal lines) and risk of several cardiovascular diseases (from logistic regression; vertical lines) for each variant used in the genetic score. The different regression lines indicate the effect sizes as calculated by different MR tests (Methods). The corresponding sensitivity analyses are provided in Supplementary Figure 2. Data sources used for these analyses, including number of individuals per trait tested, are provided in Supplementary Data 1. Abbreviations: CAD, coronary artery disease; DVT, deep vein thrombosis; LAS, large-artery stroke; IS, ischemic stroke; CES, cardioembolic stroke; SVS, small-vessel stroke; VTE, venous thromboembolism; PE, pulmonary embolism.

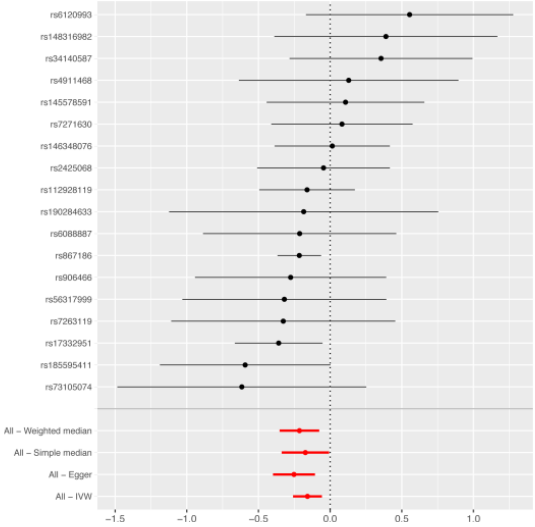

MR effect size for protein C levels on CES

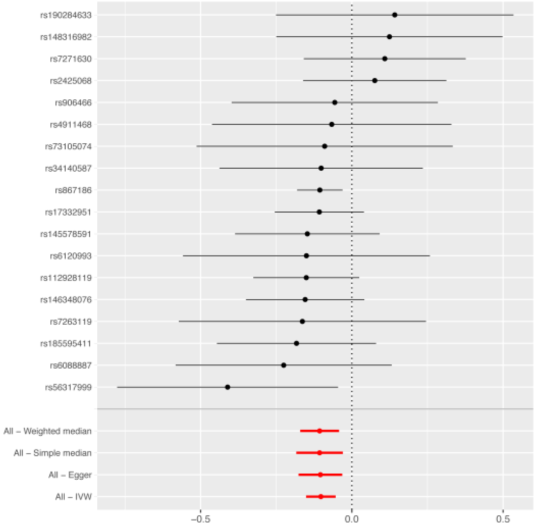

MR effect size for protein C levels on IS

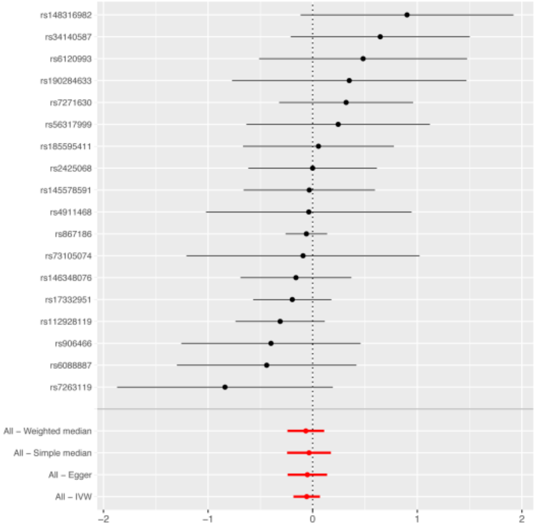

MR effect size for protein C levels on LAS

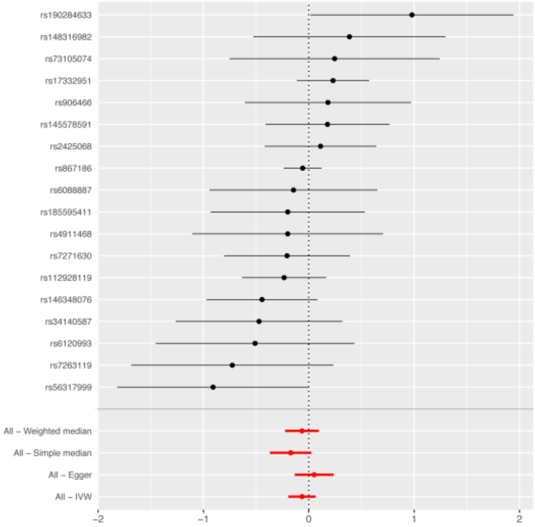

MR effect size for protein C levels on SVS

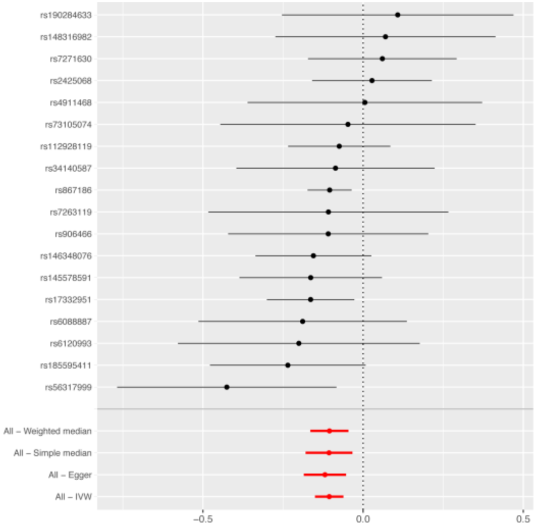

MR effect size for protein C levels on any stroke

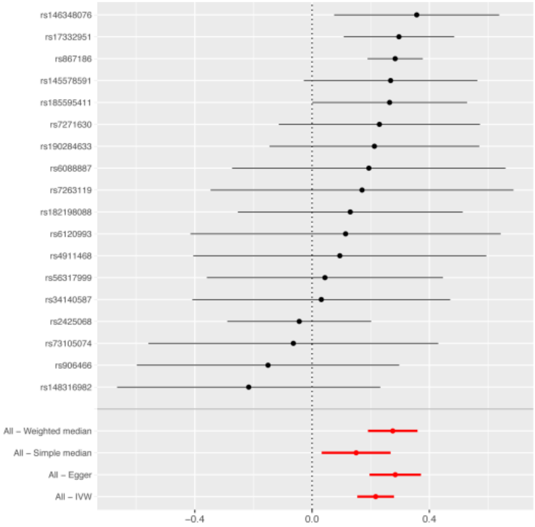

MR effect size for protein C levels on VTE

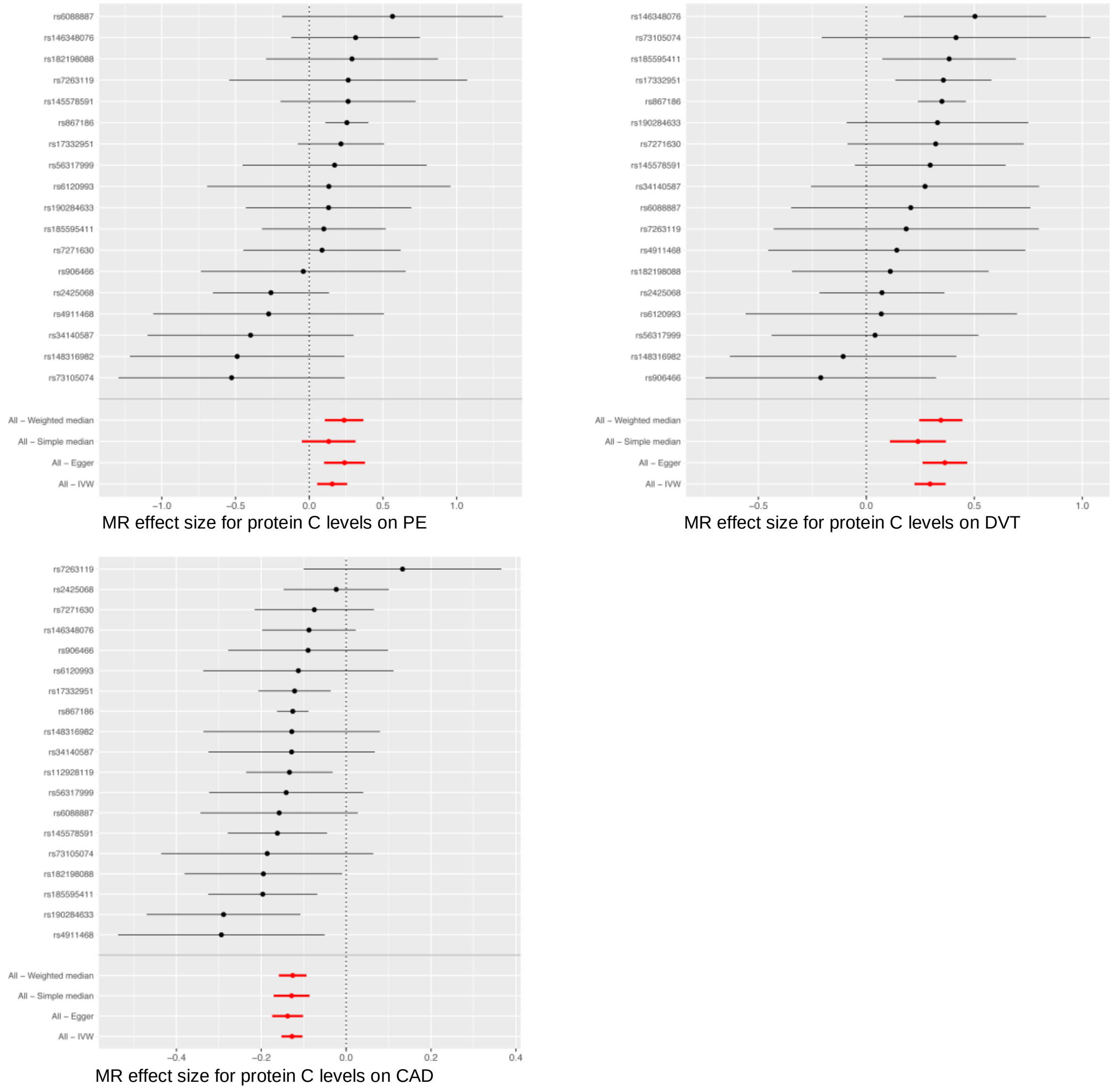


**Supplementary Figure 2. Results from Mendelian randomization analyses.** Forest plot showing the MR effect sizes and 95% confidence intervals (horizontal lines) obtained from the IVW method. Data sources used for these analyses, including number of individuals per trait tested, are provided in Supplementary Data 1. Abbreviations: LAS, large-artery stroke; IS, ischemic stroke; CES, cardioembolic stroke; SVS, small-vessel stroke; VTE, venous thromboembolism; PE, pulmonary embolism; DVT, deep vein thrombosis; CAD, coronary artery disease.

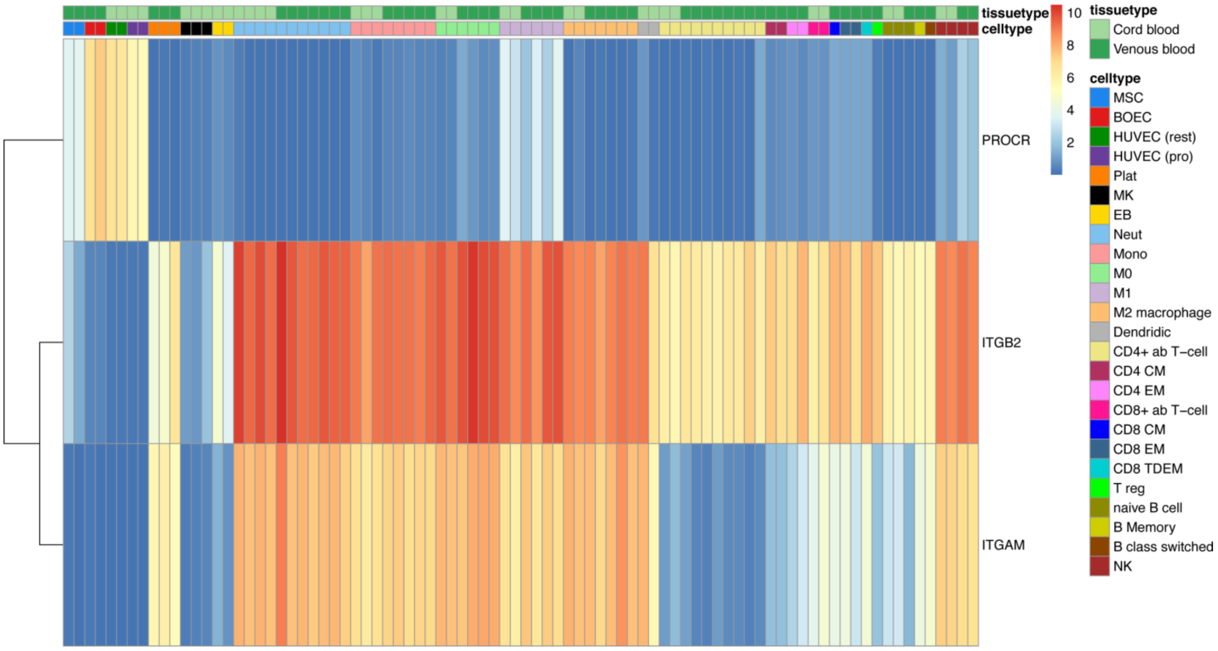


**Supplementary Figure 3. Gene expression profiles of *PROCR*, *ITGB2* and *ITGAM*.** Heatmap showing the normalized expression levels of selected candidate genes across a wide range of primary human blood cells (Grassi et al. (2021) *Haematologica.* 106(10), 2613-23). Data are shown for the *PROCR* gene (encoding the endothelial protein C receptor), *ITGAM* (CD11b) and *ITGB2* (CD18). CD11b and CD18 constitute the Macrophage-1 antigen.

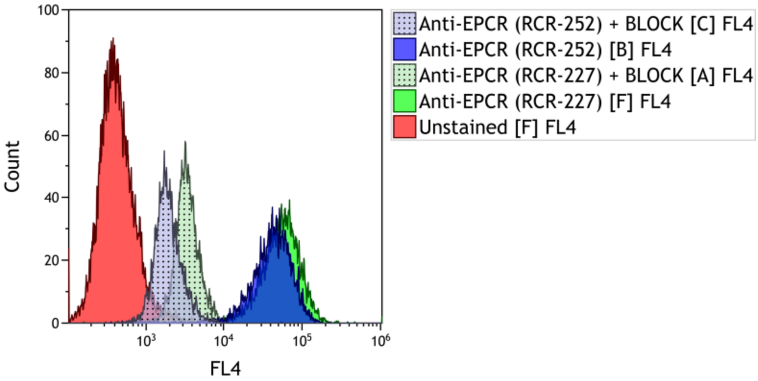


| Marker | Number | X-Med |
|---|---|---|
| All | 4,179 | 1,878.81 |
| All | 4,281 | 43,134.64 |
| All | 4,185 | 3,330.83 |
| All | 4,777 | 54,813.48 |
| All | 10,410 | 415.14 |

**Supplementary Figure 4. EPCR monoclonal antibody testing for flow cytometry.** Fluorescence intensities (FL4 channel) for human umbilical vein endothelial cells (HUVECs) stained using two allophycocyanin (APC)-conjugated EPCR monoclonal antibodies derived from distinct clones, RCR-252 and RCR-227. EPCR has been shown to be highly expressed on the surface of HUVECs (Medina et al. (2014) *Arterioscler Thromb Vasc Biol.* 34(3), 684-90). Accordingly, both antibodies yielded very high signals with median fluorescence intensities (MFI) of 43,135 and 54,813 for clones RCR-252 and RCR-227, respectively, relative to an unstained MFI of 415. To confirm the specificity of these signals, we incubated HUVECs with an unconjugated EPCR monoclonal antibody derived from the RCR-252 clone ('BLOCK') prior to the addition of an APC-conjugated antibody and subsequent flow cytometry. For both clones, we observed a marked reduction in MFI signal with a 5:1 ratio of unconjugated to APC-conjugated antibody. Taken together, these findings indicate that both of the APC-conjugated antibodies (RCR-252 and RCR-227) are specific to the EPCR protein. Abbreviations: X-Med, median fluorescence intensity.

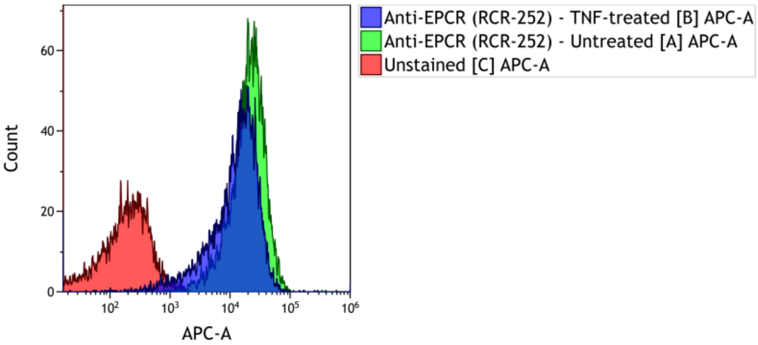


| Marker | Number | X-Med |
|---|---|---|
| All | 7,117 | 14,448.65 |
| All | 7,802 | 21,117.47 |
| All | 7,701 | 51.38 |

**Supplementary Figure 5. Quantification of surface EPCR on HUVECs.** Fluorescence intensities (APC-A channel) for HUVECs treated with 10 ng/ml tumor necrosis factor (TNF) or vehicle control and stained using an APC-conjugated EPCR monoclonal antibody derived from the RCR-252 clone. Abbreviations: X-Med, median fluorescence intensity.

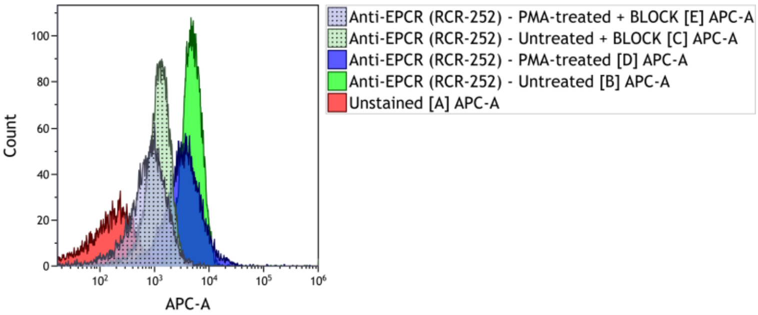


| Marker | Number | X-Med |
|---|---|---|
| All | 8,386 | 798.21 |
| All | 9,139 | 1,241.18 |
| All | 8,048 | 3,418.64 |
| All | 9,397 | 4,929.84 |
| All | 9,306 | 22.03 |

**Supplementary Figure 6. Quantification of surface EPCR on U937 cells.** Fluorescence intensities (APC-A channel) for U937 cells treated with 100 ng/ml phorbol 12-myristate 13-acetate (PMA) or vehicle control and stained using an APC-conjugated EPCR monoclonal antibody derived from the RCR-252 clone. The specificity of the vehicle control signal was assessed by pre-incubating cells with an unconjugated blocking antibody (‘BLOCK’) prior to staining. Abbreviations: X-Med, median fluorescence intensity.

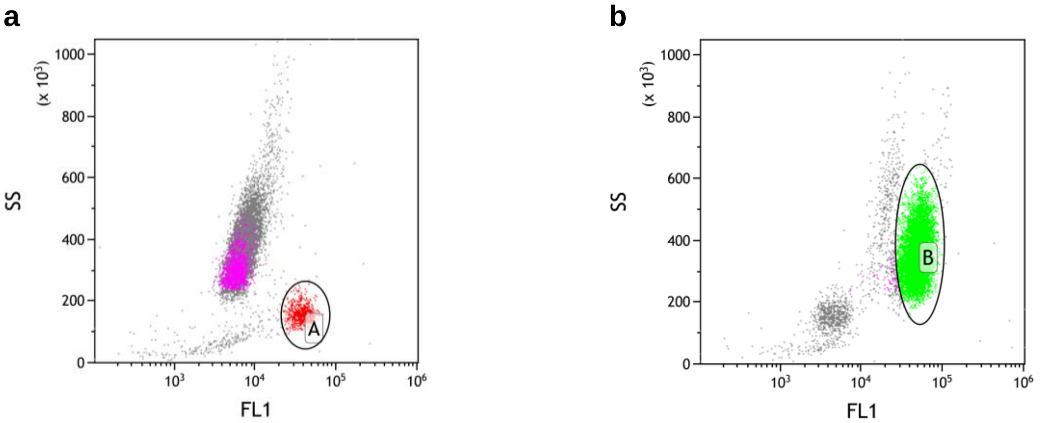


| Gate | %Gated |
|---|---|
| All | 100.00 |
| A | 6.04 |

| Gate | %Gated |
|---|---|
| All | 100.00 |
| B | 85.31 |

**Supplementary Figure 7. Gating strategy for distinguishing monocytes and neutrophils from FACS-lysed whole blood.** The gating strategy was validated using an anti-CD14 antibody for $CD14^+$ monocytes (**a**) and an anti-CD16 antibody for $CD16^+$ neutrophils (**b**). Datapoints that reflect signal from an anti-EPCR antibody are highlighted in pink.

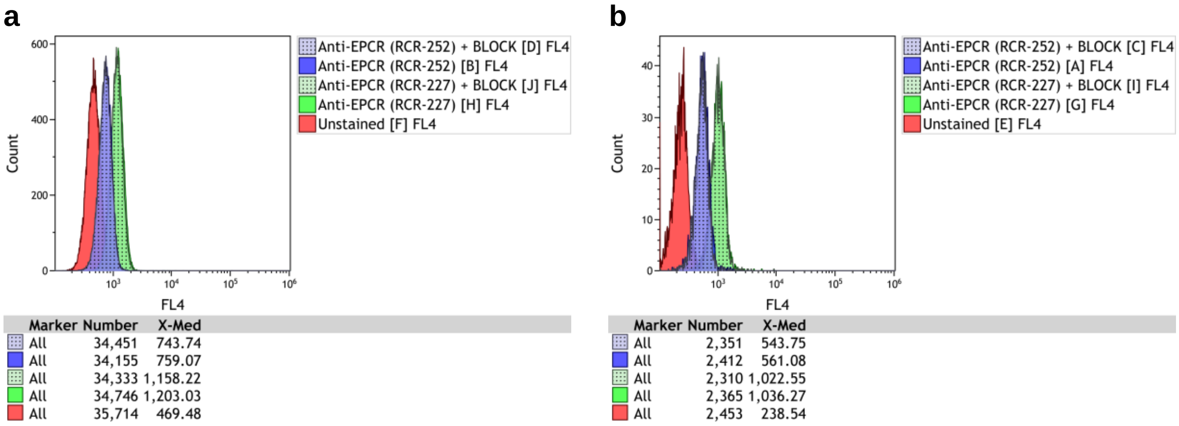


**Supplementary Figure 8. Quantification of surface EPCR on monocytes and neutrophils.** Previous studies have indicated that EPCR is expressed in other cell types beyond endothelial cells, including immune cells such as monocytes and neutrophils (Gleeson et al. (2012) *Cell Mol Life Sci.* 69(5), 717-26). To explore this, we performed flow cytometry in both primary human neutrophils (**a**) and monocytes (**b**) using two APC-conjugated EPCR monoclonal antibodies derived from distinct clones (RCR-252 and RCR-227). We observed small MFI signals (FL4 channel) of comparable magnitude for both cell types, suggesting that EPCR may be weakly expressed on the surface of monocytes and neutrophils. However, when we pre-incubated both cell types with unconjugated EPCR antibody ('BLOCK') we did not observe any reduction in signal. This indicates that the small signal observed in monocytes and neutrophils using the APC-conjugated antibodies reflects non-specific binding, suggesting that EPCR is not expressed at detectable (by flow cytometry) levels on the surface of either of these two cell types. Abbreviations: X-Med, median fluorescence intensity.

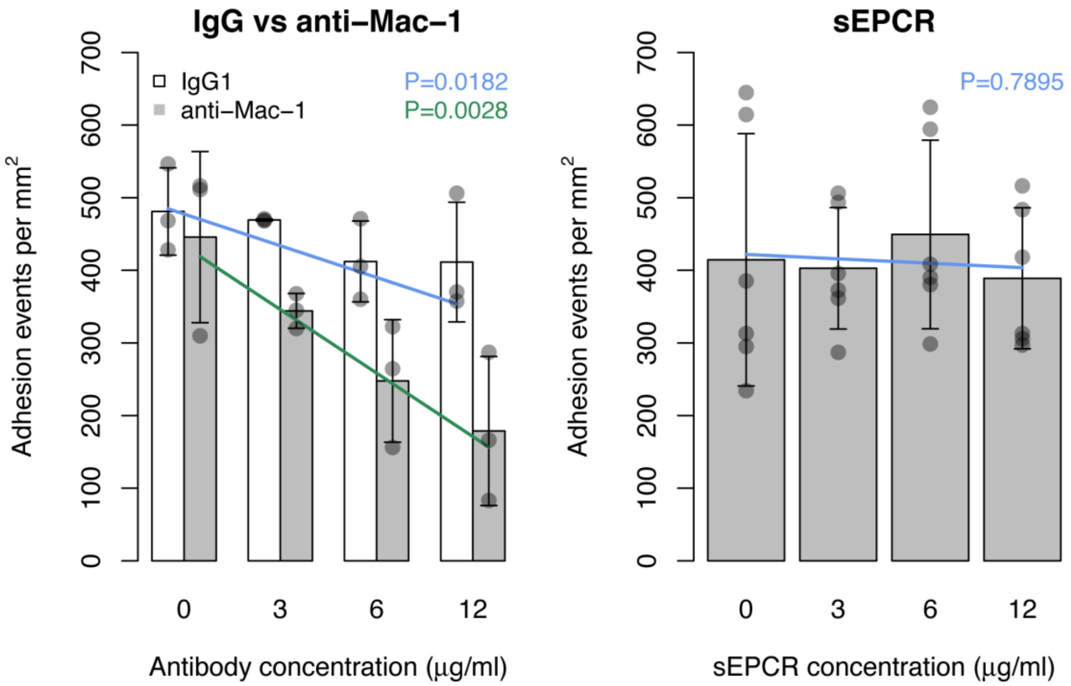


**Supplementary Figure 9. Leukocyte–endothelial cell adhesion in the presence of anti-Mac-1 antibody and sEPCR.** We performed static adhesion assays using PMA-stimulated monocytic cells (U937) and TNF-α-activated endothelial cells (HUVECs). We quantified the leukocyte–endothelial cell adhesion events after incubation with increasing concentrations of anti-Mac-1 antibody and matched IgG control, as well as recombinant sEPCR, in each separate wells of the assay (Methods). Data are shown for n=3 (IgG1), n=3 (anti-Mac-1) and n=6 (sEPCR) technical replicates. Error bars show standard deviations of the means. The blue and green lines indicate the fitted linear regression model for adhesion events~treatment concentration. To test for significance of the observed sEPCR and anti-Mac-1 effects, we used the F-test of the linear regression model. To test for significance of the IgG effect, we used a non-parametric linear model.

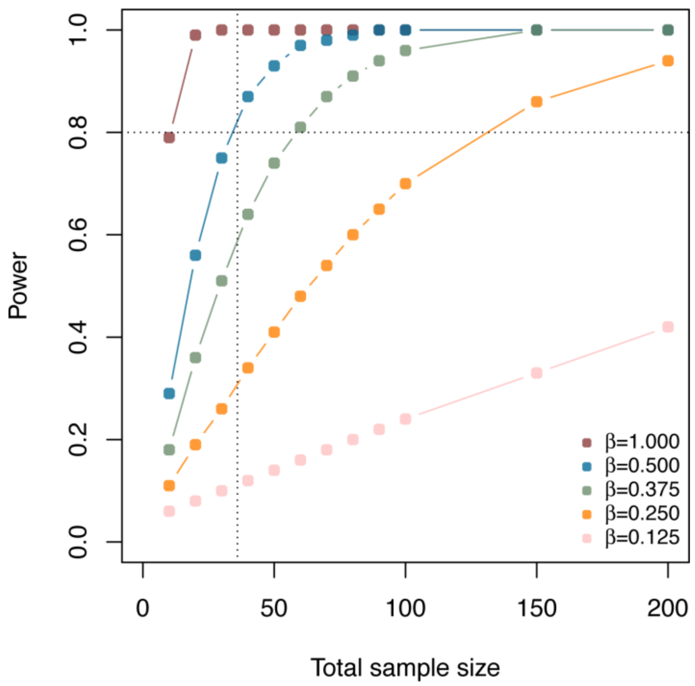


**Supplementary Figure 10. Power calculations for the recall-by-genotype study.** We compared the statistical power for varying standardized per-allele effect sizes (β) in a cohort consisting of an equal number of major and minor homozygotes of a genetic variant of interest. The calculations are independent of the allele frequency of the variant. The x-axis is the total sample size of the recall experiment; the number of eventually recruited homozygotes (n=36) is indicated with a dashed line. The y-axis is the statistical power; 80% is indicated with a dashed line. We assume a type I error rate of α=0.05. The power calculation is for an equal variance two-sample two-tailed t-test, as further described in Corbin et al. (2018) *Nat Commun.* 9(1), 711.
