# Supplementary material for: Elucidating mechanisms of genetic cross-disease associations at the PROCR vascular disease locus
Source: Nat Commun. 2022 Mar 9;13:1222. doi: 10.1038/s41467-022-28729-3 (PMC8907312; doi:10.1038/s41467-022-28729-3)
Supplement: Supplementary file 3 — Description of Additional Supplementary Files [file 41467_2022_28729_MOESM3_ESM.pdf]

SUPPLEMENTARY DATA

File Name: Supplementary Data 1

Description: Sources of contributing data for genetic analyses and association statistics. The data comprise the latest available GWAS summary statistics for a range of cardiovascular diseases and risk factors. Association statistics including effect estimates, standard errors and *P*-values were obtained from the published GWAS datasets. All data were collected from participants of European ancestry. Abbreviations: PMID, PubMed identifier; RbG, recall-by-genotype study; UKBB, UK Biobank; SE, standard error; MAF, minor allele frequency; OR, odds ratio.

File Name: Supplementary Data 2

Description: Associations of *PROCR*-rs867186 with plasma protein levels measured on the SomaScan platform. Genetic association statistics are shown for the *PROCR*-rs867186 variant for all proteins with *P*<0.05. Association statistics including effect estimates, standard errors and *P*-values were obtained from the published GWAS datasets. Data from two independent cohorts, INTERVAL (Sun et al. (2018) *Nature* 558(7708), 73-9) and KORA (Suhre et al. (2017) *Nat Commun.* 8, 14357) are shown. Data were retrieved from PhenoScanner v2 (Kamat et al. (2019) *Bioinformatics* 35, 4851-3).
