# Supplementary material for: Elucidating mechanisms of genetic cross-disease associations at the PROCR vascular disease locus
Source: Nat Commun. 2022 Mar 9;13:1222. doi: 10.1038/s41467-022-28729-3 (PMC8907312; doi:10.1038/s41467-022-28729-3)
Supplement: Supplementary file 5 — Reporting Summary [file 41467_2022_28729_MOESM5_ESM.pdf]

Corresponding author(s): Dirk S. Paul

Last updated by author(s): Jan 20, 2022

Reporting Summary

Nature Portfolio wishes to improve the reproducibility of the work that we publish. This form provides structure for consistency and transparency in reporting. For further information on Nature Portfolio policies, see our Editorial Policies and the Editorial Policy Checklist.

Statistics

For all statistical analyses, confirm that the following items are present in the figure legend, table legend, main text, or Methods section.

n/a	Confirmed	
☐	☒	The exact sample size (*n*) for each experimental group/condition, given as a discrete number and unit of measurement
☐	☒	A statement on whether measurements were taken from distinct samples or whether the same sample was measured repeatedly
☐	☒	The statistical test(s) used AND whether they are one- or two-sided<br>*Only common tests should be described solely by name; describe more complex techniques in the Methods section.*
☒	☐	A description of all covariates tested
☐	☒	A description of any assumptions or corrections, such as tests of normality and adjustment for multiple comparisons
☐	☒	A full description of the statistical parameters including central tendency (e.g. means) or other basic estimates (e.g. regression coefficient) AND variation (e.g. standard deviation) or associated estimates of uncertainty (e.g. confidence intervals)
☐	☒	For null hypothesis testing, the test statistic (e.g. *F*, *t*, *r*) with confidence intervals, effect sizes, degrees of freedom and *P* value noted<br>*Give P values as exact values whenever suitable.*
☐	☒	For Bayesian analysis, information on the choice of priors and Markov chain Monte Carlo settings
☒	☐	For hierarchical and complex designs, identification of the appropriate level for tests and full reporting of outcomes
☒	☐	Estimates of effect sizes (e.g. Cohen's *d*, Pearson's *r*), indicating how they were calculated

Our web collection on statistics for biologists contains articles on many of the points above.

Software and code

Policy information about availability of computer code

Data collection	1. PhenoScanner v2 (University of Cambridge): Data collection from published genome-wide association studies.<br>2. ImageQuant TL (GE Healthcare Life Sciences): Quantification of labeled SomaScan® aptamers.<br>3. ImagePro v6.3 (Media Cybernetics): Quantification of cell adhesion events.<br>4. Blood Atlas (University of Cambridge): Quantification of the expression levels of PROCR and related genes across a range of human blood cell types.
Data analysis	1. UK Biobank ICD PheWeb (v1.1.17): Association analyses across electronic health record-derived ICD-codes from the UK Biobank using SAIGE.<br>2. HyPrColoc (v1.0) R package: Multi-trait colocalization analyses.<br>3. PLINK v1.90: Linkage disequilibrium calculations and genetic association analyses.<br>4. MendelianRandomization (v0.3.0), TwoSampleMR (v0.3.4) and MR-PRESSO (v1.0) R packages: Mendelian randomization analyses.<br>5. R v3.4.2: Statistical analyses.<br>6. CytExpert Acquisition and Analysis Software (v2.3) (for CytoFLEX S flow cytometer), Cytomics CXP software (v2.2) (for Cytomics FC500 and Gallios flow cytometer): Acquisition of flow cytometry data.<br>7. Kaluza Analysis (v1.3): Quantification of flow cytometry data.<br>8. QuantStudio software (v1.3): Acquisition of RT-qPCR data.

For manuscripts utilizing custom algorithms or software that are central to the research but not yet described in published literature, software must be made available to editors and reviewers. We strongly encourage code deposition in a community repository (e.g. GitHub). See the Nature Portfolio guidelines for submitting code & software for further information.

Data

Policy information about availability of data

All manuscripts must include a data availability statement. This statement should provide the following information, where applicable:

- Accession codes, unique identifiers, or web links for publicly available datasets
- A description of any restrictions on data availability
- For clinical datasets or third party data, please ensure that the statement adheres to our policy

Genetic association data retrieved from the PROCR-219Gly phenome-scan are available through the UK Biobank ICD PheWeb (http://pheweb.sph.umich.edu/SAIGE-UKB/variant/20:33764554-A-G) and PhenoScanner (http://www.phenoscanner.medschl.cam.ac.uk/?query=rs867186&catalogue=GWAS&p=5e-8&proxies=None&r2=0.8&build=37/). GWAS summary statistics at the PROCR locus used for colocalization and Mendelian randomization analyses are available as follows: stroke (Malik 2018; https://www.ebi.ac.uk/gwas/publications/29531354), CAD (van der Harst 2017; https://www.ebi.ac.uk/gwas/publications/29212778) and APC (Sun 2018; https://www.ebi.ac.uk/gwas/publications/29875488) data are available for FTP download from the NHGRI-EBI Catalog of GWAS. FVII and PC data (Tang 2010) are available on request from the ARIC study at: https://sites.cscc.unc.edu/aric/distribution-agreements. Pulmonary embolism (phenotype ID: 20002_1093), VTE (I9_VTE) and DVT (20002_1094) data can be downloaded from the UK Biobank (http://www.nealelab.is/uk-biobank) using the following wget commands: Pulmonary embolism: 'wget https://broad-ukb-sumstats-us-east-1.s3.amazonaws.com/round2/additive-tsvs/20002_1093.gwas.imputed_v3.both_sexes.tsv.bgz -O 20002_1093.gwas.imputed_v3.both_sexes.tsv.bgz'; VTE: 'wget https://broad-ukb-sumstats-us-east-1.s3.amazonaws.com/round2/additive-tsvs/I9_VTE.gwas.imputed_v3.both_sexes.tsv.bgz -O I9_VTE.gwas.imputed_v3.both_sexes.tsv.bgz'; DVT: 'wget https://broad-ukb-sumstats-us-east-1.s3.amazonaws.com/round2/additive-tsvs/20002_1094.gwas.imputed_v3.both_sexes.tsv.bgz -O 20002_1094.gwas.imputed_v3.both_sexes.tsv.bgz'. Suppl. Table 1 provides further information on the genetic data sources. All other data that support the findings of this study are available from the corresponding author upon reasonable request.

Field-specific reporting

Please select the one below that is the best fit for your research. If you are not sure, read the appropriate sections before making your selection.

☒ Life sciences ☐ Behavioural & social sciences ☐ Ecological, evolutionary & environmental sciences

For a reference copy of the document with all sections, see nature.com/documents/nr-reporting-summary-flat.pdf

Life sciences study design

All studies must disclose on these points even when the disclosure is negative.

Sample size

For the recall-by-genotype study, we recruited all available healthy volunteers from the NIHR Cambridge BioResource who are carriers of the PROCR-rs867186 minor allele; met the pre-specified inclusion criteria; and agreed to participate in the study. We then matched rs867186 major allele carriers and heterozygotes accordingly. For sample size calculations, we also applied the webtool 'Recall by Genotype Study Planner' (https://timpsonlab-mrc-ieu.shinyapps.io/rbgstudyplanner/).

For the in vitro experiments, we used between 4-5 biological replicates for each condition, along with multiple technical replicates. We also incorporated a gradient design where feasible (e.g. RT-qPCR). The sample sizes are in accordance with standard practices for in vitro experiments, and were recommended and approved during the peer-review process.

Data exclusions

Data of biomarker levels from participants that were 3 standard deviations above or below the population mean were excluded from analyses. This exclusion criterion was pre-established to reduce the potential for confounding due to unmeasured variables.

Replication

We generated biomarker data in healthy volunteers through our recall-by-study under the strict control of experimental conditions. These data serve as a replication of the biomarker data generated for large-scale genome-wide association studies, for which the experimental conditions cannot be controlled to the same extent. For both soluble EPCR (sEPCR) and protein C (PC), our recall-study serve as replication concerning both significance and directionality for findings from previously published studies, as noted in the manuscript. The replication for PC is robust to different measurement assays (i.e. immunoassays and chromogenic assays). For APC, in our recall-study (immunoassay), we were unable to replicate the SomaScan data (Sun, 2018) reported in our phenome-scan; however, as noted in the Discussion section, this was likely due to the enhanced statistical power of the SomaScan study.

In our in vitro experiments, our findings relating to membrane-bound EPCR serve as replication for previously published in vitro studies (PMIDs: 24436369; 16409473). For RT-qPCR and adhesion assay findings, we provided cross-cell-type replication, with concordant results shown in two types of endothelial cells; human umbilical vein endothelial cells (HUVECs) and human coronary artery endothelial cells (HCAECs). These findings are also in agreement with previously published studies (PMIDs: 11278252; 15505101), although these data were generated using different cellular models and treatment conditions.

Randomization

In our recall-study, participants who were older than 18 years of age and of European ancestry were selected based on PROCR-rs867186 genotype and homozygosity of the major allele for both F5-R506Q (rs6025; Factor V Leiden) and F2-G20210A (rs1799963; Factor II). Participants across the three rs867186 genotype groups were matched at the end of the study with respect to sex and age (within 10 years). Study participants were excluded that had a diagnosis of (i) a chronic disease; (ii) hypertension (or history of consistently high blood pressure readings, i.e. >140/90 mmHg); and/or (iii) hypercholesterolemia (or history of consistently high cholesterol levels, i.e. >6 mmol/l).

For our in vitro experiments, cell lines were either selected based solely on PROCR-rs867186 genotype status or were selected by the supplier (in our case, Lonza). For experiments that did not involve stratification by genotype (i.e. RT-qPCR and adhesion assays), we adopted a within-subject design.

Blinding

For our recall-study, all investigators were blinded to the genotype status of the volunteers at both the data collection and analysis stages. As the in vitro experiments were performed by a single investigator, the investigator was not blinded to group allocation, but where possible,

efforts were made to validate quantifications and analyses by a blinded investigator (e.g. quantification of cell adhesion events).

Reporting for specific materials, systems and methods

We require information from authors about some types of materials, experimental systems and methods used in many studies. Here, indicate whether each material, system or method listed is relevant to your study. If you are not sure if a list item applies to your research, read the appropriate section before selecting a response.

Materials & experimental systems

n/a	Involved in the study
☐	☒ Antibodies
☐	☒ Eukaryotic cell lines
☒	☐ Palaeontology and archaeology
☒	☐ Animals and other organisms
☐	☒ Human research participants
☒	☐ Clinical data
☒	☐ Dual use research of concern

Methods

n/a	Involved in the study
☒	☐ ChIP-seq
☐	☒ Flow cytometry
☒	☐ MRI-based neuroimaging

Antibodies

Antibodies used

1. APC-conjugated rat anti-human EPCR monoclonal antibody (Supplier: BD Biosciences; Cat. No: 563622; Clone: RDR-252)
2. APC-conjugated rat anti-human EPCR monoclonal antibody (Supplier: ThermoFisher Scientific; Cat. No: 17-2018-42; Clone: RCR-227)
3. APC-conjugated rat IgG1, K isotype control antibody (Supplier: BD Biosciences; Cat. No: 554686; Clone: R3-34)
4. APC-conjugated rat IgG1, K isotype control antibody (Supplier: ThermoFisher Scientific; Cat. No: 17-4301-82; Clone: eBRG1)
5. Unconjugated rat anti-human EPCR monoclonal antibody (Supplier: BD Biosciences; Cat. No: 552500; Clone: RCR-252)
6. Unconjugated mouse anti-human CD11b/Integrin alpha M antibody (anti-Mac1) (Supplier: BioTechne; Cat. No: MAB1699; Clone: ICRF44)
7. FITC-conjugated mouse anti-human CD16 antibody (Supplier: BioLegend; Cat. No: 360715; Clone: B73.1)
8. FITC-conjugated mouse anti-human CD14 antibody (Supplier: BioLegend; Cat. No: 325603; Clone: HCD14)

We have indicated the final concentrations of each antibody used in the flow cytometry subsections of the Methods section. We chose to provide final concentrations rather than dilutions because (1) the concentrations of some of the antibodies can differ by Lot. No., and (2) the isotype control antibodies were supplied at a different stock concentration relative to the experimental antibodies.

Validation

Details describing antibody #1 can be found at: https://www.bdbiosciences.com/us/applications/research/stem-cell-research/cancer-research/human/pe-rat-anti-human-cd201-rcr-252/p/557950. The antibody has been used in two previous studies in either flow cytometry or FACS (PMIDs: 11246560; 11686350) and was tested by the manufacturers in ECV304 cells. Details describing antibody #2 can be found at: https://www.thermofisher.com/antibody/product/CD201-EPCR-Antibody-clone-RCR-227-Monoclonal/17-2018-42. The antibody has been used in two previous studies in either flow cytometry or FACS (PMIDs: 26053033; 29217770) and was tested by the manufacturers in flow cytometry of HUVECs. We also validated the specificity of both antibodies by pre-incubating HUVECs with unconjugated EPCR antibodies prior to staining with the APC-conjugated antibodies (Suppl. Fig. 4 of the manuscript).

Details describing antibody #1 can be found at: https://www.bdbiosciences.com/us/applications/research/stem-cell-research/cancerresearch/human/pe-rat-anti-human-cd201-rcr-252/p/557950. The antibody has been used in two previous studies in either flow cytometry or FACS (PMIDs: 11246560; 11686350) and was tested by the manufacturers in ECV304 cells.

Details describing antibody #2 can be found at: https://www.thermofisher.com/antibody/product/CD201-EPCR-Antibody-clone-RCR-227-Monoclonal/17-2018-42. The antibody has been used in two previous studies in either flow cytometry or FACS (PMIDs: 26053033; 29217770) and was tested by the manufacturers in flow cytometry of HUVECs. We also validated the specificity of both antibodies by pre-incubating HUVECs with unconjugated EPCR antibodies prior to staining with the APC-conjugated antibodies (Suppl. Fig. 4 of the manuscript).

Antibodies #3 and #4 were the recommended isotype controls for antibodies #1 and #2, respectively. Details describing antibody #3 can be found at: https://www.bdbiosciences.com/en-gb/products/reagents/flow-cytometry-reagents/research-reagents/flow-cytometry-controls-and-lysates/apc-rat-igg1-isotype-control.554686. Details for antibody #4 can be found at: https://www.thermofisher.com/antibody/product/Rat-IgG1-kappa-clone-eBRG1-Isotype-Control/17-4301-82.

Details describing antibody #5 can be found at: https://www.bdbiosciences.com/en-gb/products/reagents/flow-cytometry-reagents/research-reagents/single-color-antibodies-ruo/purified-rat-anti-human-cd201.552500. The antibody has been validated in flow cytometry in ECV304 cells, and has been used in previously published studies (PMIDs: 11686350; 12714492; 11246560).

Details describing antibody #6 can be found at: https://www.bio-techne.com/p/antibodies/human-cd11b-integrin-alpha-m-antibody-icrf44_mab1699. The antibody has been validated for flow cytometry and immunocytochemistry, and has been used in previously published studies (PMIDs: 20536386; 20220088; 15147571).

Details describing antibody #7 can be found at: https://www.biolegend.com/en-gb/products/fitc-anti-human-cd16-antibody-9302?GroupID=BLG12171. The antibody has been validated for flow cytometry in human peripheral blood lymphocytes, and has been used in previously published studies (PMIDs: 27595670; 28785262; 32385277).

Details describing antibody #8 can be found at: https://www.biolegend.com/en-gb/products/fitc-anti-human-cd14-antibody-3951. The antibody has been validated for flow cytometry in human peripheral blood monocytes, and has been used in previously published studies (e.g. PMIDs: 29290585; 29861170; 29491406).

Eukaryotic cell lines

Policy information about cell lines

Cell line source(s)	1. U-937 (Supplier: ATCC; Cat. No: CRL-1593.2)<br>2. Human umbilical vein endothelial cells (HUVECs), pooled, in EGM-2 (Supplier: Lonza; Cat. No: C2519A)<br>3. Human coronary artery endothelial cells (HCAECs) (Supplier: Lonza; Cat. No: CC-2585)<br>4. Genotype-specific HUVECs were obtained from the Anthony Nolan Trust Biobank (University of Leicester, UK)
Authentication	None of the cell lines used was authenticated.
Mycoplasma contamination	U937 cells, HUVECs and HCAECs tested negative for mycoplasma contamination prior to use.
Commonly misidentified lines (See ICLAC register)	None of the cell lines used in this study is listed in the Register of Misidentified Cell Lines.

Human research participants

Policy information about studies involving human research participants

Population characteristics	Participants who were older than 18 years of age and of European ancestry were selected based on PROCR-rs867186 genotype and homozygosity of the major allele for both F5-R506Q (rs6025; Factor V Leiden) and F2-G20210A (rs1799963; Factor II). Study participants were excluded that had a diagnosis of (i) a chronic disease; (ii) hypertension (or history of consistently high blood pressure readings, i.e. >140/90 mmHg); and/or (iii) hypercholesterolemia (or history of consistently high cholesterol levels, i.e. >6 mmol/l). Participants agreed to fast and abstain from caffeinated drinks for at least four hours prior to the study visit and to not receive any vasoactive medication for up to seven days prior to procedures. The percentage of volunteers that were female was 32.6% and the mean age was 49.7 years. A summary of the covariate-relevant population characteristics, including body mass index (BMI), heart rate and blood pressure, are summarised in Suppl. Table 6. We did not identify statistically significant differences between the genotypic groups for any of these characteristics (Suppl. Table 6).
Recruitment	Healthy volunteers were recruited from the NIHR Cambridge BioResource with informed consent. All volunteers were living in Cambridgeshire and were blood donors who had consented for re-contact. None of these factors is expected to have impacted the validity of our results from the recall-study. This statement is supported by the high concordance between our findings for sEPCR and PC and those from previously published studies.
Ethics oversight	The study was approved by the Leicester Central Research Ethics Committee and Health Research Authority (Reference: 17/EM/0028).

Note that full information on the approval of the study protocol must also be provided in the manuscript.

Flow Cytometry

Plots

Confirm that:

- [x] The axis labels state the marker and fluorochrome used (e.g. CD4-FITC).
- [x] The axis scales are clearly visible. Include numbers along axes only for bottom left plot of group (a 'group' is an analysis of identical markers).
- [x] All plots are contour plots with outliers or pseudocolor plots.
- [x] A numerical value for number of cells or percentage (with statistics) is provided.

Methodology

Sample preparation	For flow cytometry analyses of primary human monocytes and neutrophils in whole blood, we lysed 100-µl citrated whole-blood samples for 10 min at room temperature using Lysing Solution 10X Concentrate (349202; BD Biosciences). Lysed blood was then centrifuged at 600×g for 6 min and 4°C, and the pellet re-suspended in HEPES buffered saline (Sigma-Aldrich). Cultured cells were also re-suspended in HEPES buffered saline, to a final concentration of 10^5 cells/100 µl.
Instrument	We used the following flow cytometers: Cytomics FC500, CytoFLEX S and Gallios (all Beckman Coulter).
Software	Flow cytometry data were acquired using the Cytomics CXP software (v2.2) (for Cytomics FC500 and Gallios flow cytometer) and CytExpert Acquisition and Analysis Software (v2.3) (for CytoFLEX S flow cytometer). Downstream analyses were performed using Kaluza Analysis (v1.3).

Cell population abundance	Cell population abundance was not determined.
Gating strategy	In flow cytometry of whole-blood samples, we gated using forward vs. side scatter (FSC vs. SSC) to identify monocyte (agranular, large) and neutrophil (granular) populations. We validated our gating strategy using CD14 and CD16 antibodies as markers of monocytes and neutrophils, respectively. Suppl. Fig. S7 provides an overview of the gating strategy.

- [x] Tick this box to confirm that a figure exemplifying the gating strategy is provided in the Supplementary Information.
